# Coexistence of Tuberculosis and Lophomoniasis in a Patient With Alzheimer's Disease

**DOI:** 10.1002/rcr2.70248

**Published:** 2025-06-25

**Authors:** Masoud Maboudi, Eissa Soleymani, Elham Sadat Banimostafavi, Shirafkan Kordi, Majidreza Adelani, Zakaria Zakariaei, Mahdi Fakhar

**Affiliations:** ^1^ Department of Infectious Diseases, Faculty of Medicine, Antimicrobial Resistance Research Center Mazandaran University of Medical Sciences Sari Iran; ^2^ Toxoplasmosis Research Center, Communicable Diseases Institute, Department of Parasitology Mazandaran University of Medical Sciences Sari Iran; ^3^ Department of Parasitology and Mycology, Faculty of Medicine Mazandaran University of Medical Sciences Sari Iran; ^4^ Department of Radiology Shahid Beheshti Hospital, Qom University of Medical Sciences Qom Iran; ^5^ Department of Internal Ward, Pulmonary and Critical Care Division Mazandaran University of Medical Sciences Sari Iran; ^6^ Department of Forensic Medicine and Toxicology, Mazandaran Registry Center for Opioids Poisoning, Orthopedic Research Centers Imam Khomeini Hospital, Mazandaran University of Medical Sciences Sari Iran; ^7^ Iranian National Registry Centre for Lophomoniasis and Toxoplasmosis, Imam Khomeini Hospital Mazandaran University of Medical Sciences Sari Iran; ^8^ Department of Medical Microbiology and Immunology, School of Medicine Qom University of Medical Sciences Qom Iran

**Keywords:** Alzheimer's disease, *Lophomonas*, Lophomoniasis, metronidazole, *Mycobacterium*

## Abstract

The coexistence of lophomoniasis and tuberculosis (TB), both airborne diseases, is relatively uncommon. Co‐infections like these can complicate treatment strategies due to overlapping symptoms and potential drug interactions. We report a rare case of comorbidity involving two pulmonary diseases, lophomoniasis and TB, in an 82‐year‐old woman with Alzheimer's disease (AD) from northern Iran. Her primary symptoms included weakness, lethargy, dyspnea, sputum production, night sweats, and significant weight loss. Both TB and lophomoniasis can compromise the immune system, potentially worsening the progression or severity of AD by increasing susceptibility to infections or enhancing neuroinflammation. Following the prescription of appropriate drug regimens for both diseases, the patient was discharged from the hospital in stable condition. Overall, it is crucial to consider lophomoniasis in the differential diagnosis of patients with pulmonary tuberculosis, especially in endemic areas where both infections are prevalent, to ensure timely diagnosis and effective management.

## Graphical abstract

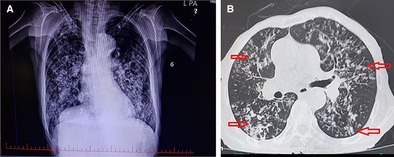

## 1 Introduction

Tuberculosis (TB) is an infection with a long history, having impacted human populations for more than 4000 years [1]. In 2020, TB was responsible for the deaths of approximately 1.5 million individuals, which included 214,000 people living with HIV. Also, it ranks as the 13th leading cause of death worldwide due to infectious diseases [2]. The disease predominantly affects various organ systems, including the respiratory, gastrointestinal, lymphoreticular, central nervous, integumentary, musculoskeletal, reproductive, and hepatic systems [3]. Pulmonary TB is characterised by a range of clinical symptoms, such as a persistent cough, production of sputum, decreased appetite, unintentional weight loss, fever, night sweats, and hemoptysis [4].

Lophomoniasis is an emerging neglected parasitic disease attributed to the *Lophomonas blattarum* (*L. blattarum*) [5]. It is a flagellated protozoan pathogen classified within the order Hypermastigidia and the suborder Lophomonadia that inhabits the hindgut of several arthropod species, including termites and cockroaches [6]. Nevertheless, this neglected protozoan has the potential to act as an emerging pathogen, capable of infecting human hosts, predominantly via the upper and lower respiratory tracts [2]. In humans, it has the capacity to invade upper and lower respiratory tracts; even it may lead to severe and potentially irreversible complications, such as the formation of a pulmonary cavity [7]. The predominant symptoms include fever, chronic cough, and mucus production, which can escalate to respiratory failure. Pneumonia, bronchiectasis, lung abscesses, and pleural effusions may occur due to this pathogen. Consequently, due to clinical findings and laboratory tests, differentiating these symptoms from other prevalent conditions that exhibit similar manifestations, such as pneumonia and bronchitis, poses a significant challenge [8].

Currently, microscopic examination of the bronchoalveolar lavage (BAL) specimen is routinely performed for diagnosing lophomoniasis in diagnostic laboratories by expert personnel. Due to its resemblance to atypical respiratory epithelial cells, accurate detection of this parasite using a light microscope is challenging, often resulting in it being overlooked in diagnostic evaluations [6]. Based on the findings from a registry‐based clinical study in Iran, pulmonary lophomoniasis does not exhibit pathognomonic bronchoscopic findings. This suggests that the bronchoscopic appearance of this condition can vary significantly and may not provide definitive diagnostic clues. However, the study highlighted that the right lung bronchus was observed to have the highest frequency of abnormal views during bronchoscopic examinations [9].

Numerous reports have presented the comorbidity of *L. blattarum* and *Mycobacterium* [2, 10, 11]. In this report, we detail a rare case of comorbidity involving lophomoniasis and TB, in an old woman with Alzheimer's disease (AD).

## 2 Case Report

In October 2024, an 82‐year‐old woman, a known case of AD, was admitted to Razi Hospital in Qaemshahr, Mazandaran province, northern Iran. She presented with symptoms including weakness, lethargy, dyspnea, sputum production, and night sweats, alongside a notable weight loss of 10 kg over the course of a year. The patient's vital signs recorded in the emergency ward were as follows: blood pressure measured at 95/60 mmHg, temperature at 36.9°C, respiratory rate at 18 breaths per minute, radial pulse at 90 beats per minute, and peripheral oxygen saturation (SO_2_) at 84% on room air.

**FIGURE 1:**
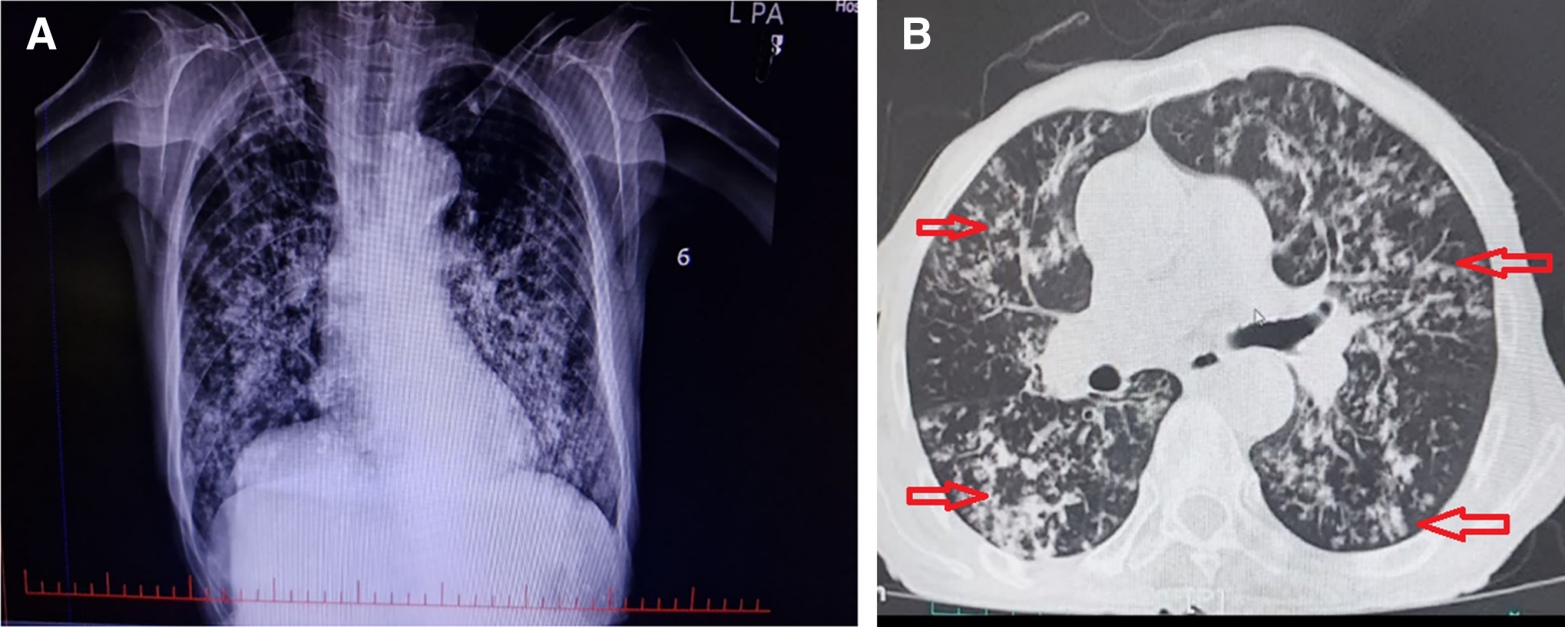
(A) A chest X‐ray showing alveolar opacities in both the lungs; (B) An axial lung CT‐scan showing reduction of volume in the right lung. Additionally, multiple centrilobular nodules with peripheral tree‐in‐bud pattern (red arrows), scattered in both the lungs.

The patient was immunocompetent with a history of hypertension. Within imaging worked up, a chest X‐ray showed alveolar opacities in both the lungs predominantly in the right middle and left lower lobes (Figure 1A). Furthermore, the patient received a chest‐computed tomography (CT) scan, which revealed the presence of cavitary lesions accompanied by a reduction in lung parenchymal volume within the right upper lobe. Additionally, centrilobular nodules exhibiting a linear branching configuration, often described as a “tree in bud” pattern, were observed in both the lungs periphery (Figure 1B). Furthermore, bronchoscopic findings showed a slightly narrow trachea, an expanded carina, and mild mucosal edema and hyperemia in the right bronchus.

The lab tests indicated an abnormality in levels of erythrocyte sedimentation rate (ESR) (65 mm/h), C‐reactive protein (CRP) (42 mg/L), lactate dehydrogenase (LDH) (1520 U/L), blood urea nitrogen (BUN) (104 mg/dL) and creatinine (Cr) (1.3 mg/dL). Other laboratory items were normal.

Because of patient disability for giving sputum sample, a BAL fluid (BALF) acid‐fast bacillus (AFB) smear test and real‐time PCR were conducted in accordance with the patient's clinical presentation and the prevalence of TB. BAL sample showed a direct smear positive for AFB with a grading of 3+. Consequently, the patient was prescribed a fixed‐dose combination (3FDC) regimen consisting of isoniazid (75 mg), rifampin (150 mg), pyrazinamide (400 mg), and ethambutol (275 mg) for a duration of 1 week. The patient's BALF AFB smear was monitored by real‐time PCR monthly until it returned negative after 8 weeks of treatment, as per our institutional protocols.

**FIGURE 2:**
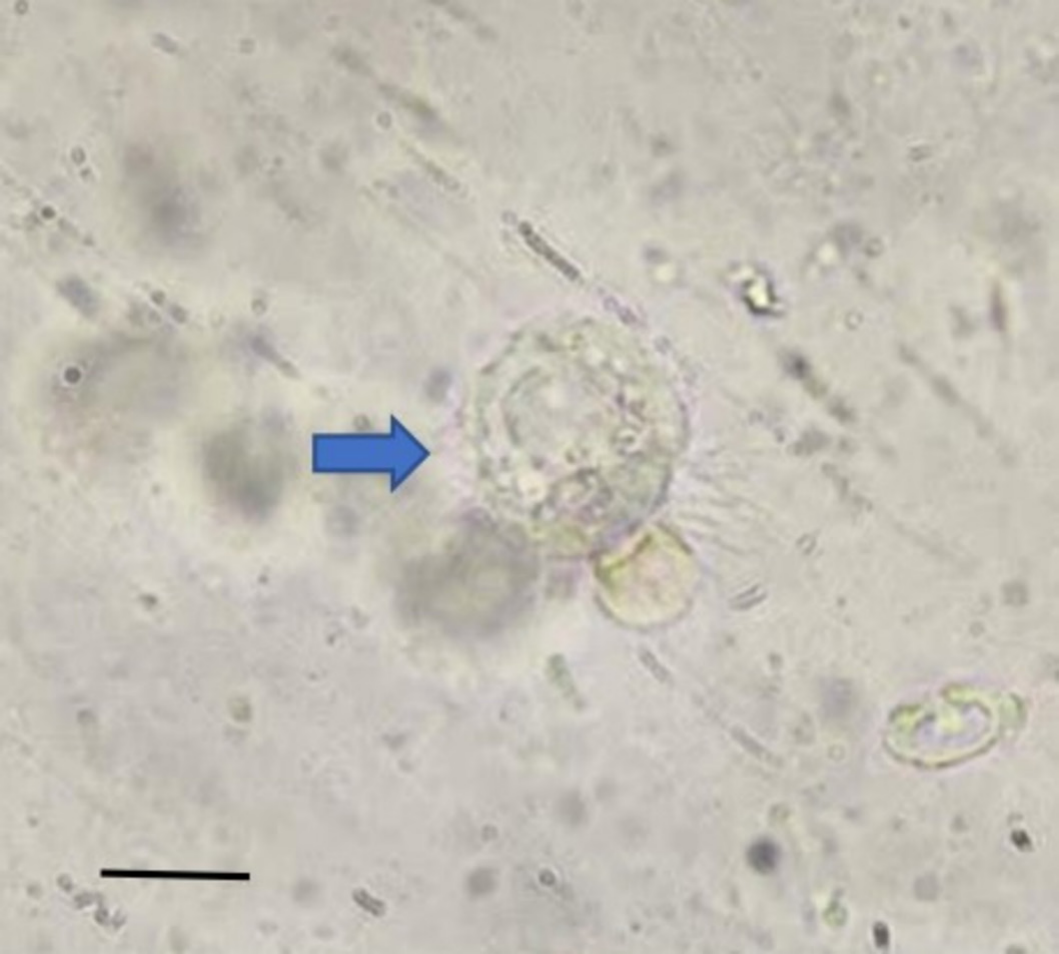
A wet smear showing a trophozoite of *Lophomonas* pathogen in BALF specimen (arrow head) (400×). Bar = 10 nm.

Moreover, the initial BALF sample was examined for further laboratory analysis. This BALF sample was utilised to identify *Lophomonas* infection. Lophomoniasis was diagnosed through microscopic evaluation via wet smear technique. Observation by light microscope showed a pleomorphic and motile multiflagellated protozoan trophozoite characterised by granular cytoplasm and the absence of a distinct nucleus, confirming the presence of *Lophomonas* spp. (see Figure 2 and Video 1). According to our experience at the Iranian National Registry Center for Lophomoniasis (INRCL), a patient's parasite severity index was classified as severe, indicating 1–10 parasites per high‐power microscopic field [6]. No fungal and bacterial pathogens were detected in the BALF specimen.

**VIDEO 1:** A motile tufted *Lophomonas* trophozoite in a BAL sample. Video content can be viewed at https://onlinelibrary.wiley.com/doi/10.1002/rcr2.70248

Following the diagnosis of this infection, the patient was prescribed oral metronidazole (500 mg twice daily) for 2 weeks. Following treatment, the patient's symptoms completely resolved by the 6‐month follow‐up visit and the patient reported no further complaints. However, despite our requests, she and her family declined to undergo a chest X‐ray or CT scan. The diagnosis of true co‐infection in our patient was confirmed through a combination of imaging and bronchoscopic findings related to both pathogens. This was further supported by clinical correlation, standard laboratory confirmation and severity index, and ultimately by successful treatment outcomes.

The presentation of this research adheres to the CARE guidelines. We obtained the patient's consent prior to initiating treatment for lophomoniasis and tuberculosis, as well as conducting other diagnostic procedures as per our institutional protocols.

## 3 Discussion

In this report, we present the case of a patient with AD co‐infected with the *Lophomonas* pathogen and *Mycobacterium tuberculosis* . Understanding co‐infections involving TB is crucial in regions where these diseases are endemic, as it helps develop targeted public health interventions that address multiple pathogens simultaneously. While lophomoniasis is often viewed as a benign or self‐limiting infection in immunocompetent individuals, our patient presented with additional comorbidities that warranted a more aggressive treatment approach. These comorbidities, coupled with the severity of the infection as indicated by the patient's symptoms and laboratory findings, guided our decision to extend the treatment duration. Furthermore, the literature suggests that in certain cases, especially when complications or coexisting conditions are present, a longer treatment course may enhance recovery and reduce the risk of potential recurrence [5, 12]. Thus, we believed that this tailored approach was in the best interest of the patient's overall health and well‐being.

The coexistence of TB and lophomoniasis in a patient with AD is of particular notice for several reasons: TB induces a complex inflammatory response, which is believed to play a critical role in the development of AD. Although in cases where AD is already clinically established at the time of TB diagnosis, the causal relevance of TB‐induced inflammation to AD development diminishes. Proinflammatory cytokines such as TNFα, IL‐6, IFN‐γ, and IL‐1β are involved in both TB and Alzheimer's pathogenesis [13]. The presence of another infection like lophomoniasis could exacerbate this inflammatory state. Both TB and lophomoniasis can compromise the immune system, potentially worsening the progression or severity of AD by increasing susceptibility to infections or enhancing neuroinflammation. Co‐infections like these can complicate treatment strategies due to overlapping symptoms and potential drug interactions. Managing multiple infections simultaneously requires careful consideration to avoid adverse effects while ensuring effective treatment for each condition [14, 15].

In our patient, the elevation of LDH, CRP, and ESR was noted. In pulmonary infections like TB and lophomoniasis, increased LDH could reflect lung tissue involvement or inflammation. The presence of elevated CRP suggests active inflammation due to either TB or lophomoniasis, which could exacerbate neuroinflammation associated with AD. High ESR values alongside other inflammatory markers indicate ongoing systemic inflammation that might complicate management by increasing susceptibility to further infections or worsening neurodegenerative processes. Interpreting these values collectively provides insight into the patient's overall inflammatory status and potential severity of their conditions. Monitoring these markers during treatment can help assess how effectively the patient is responding to therapy for both infections and guide adjustments if necessary. Overall, understanding these biomarkers' elevations helps clinicians tailor treatment strategies for managing complex comorbidities like those presented in this case.

The primary treatment for lophomoniasis is metronidazole, with dosing regimens adjusted based on patient age and infection severity. For adults, the standard dose is 500–750 mg every 8 h, administered orally or intravenously, typically for 7–14 days [2, 5, 7, 12]. Paediatric patients are treated with 30–40 mg/kg/day divided into three doses, often extending to 3 weeks. Alternative therapies include tinidazole (500 mg every 12 h for 7–14 days) and albendazole (400 mg/day for 5 days), though metronidazole remains the first‐line treatment due to its high efficacy and absence of reported resistance [16]. However, significant variability in dosing regimens exists across studies and clinical cases, underscoring the lack of established, standardised treatment guidelines. Clinical improvement, including resolution of symptoms such as cough and fever, is commonly observed post‐treatment [12].

Our patient lived in urban areas of northern Iran, and the people living there are more exposed to cockroaches and subsequently *Lophomonas* infection due to the high population of these insects [6]. Spending more time indoors may lead to greater exposure to domestic insects like cockroaches and termites, as well as dust infected with these insects [6]. On the other hand, cockroaches are considered the main reservoir host for this parasite. As a result, those who live in urban areas may be more at risk [6]. In this regard, Fakhar et al. claimed that the parasite mainly affects immunocompetent individuals rather than immunocompromised ones, and *Lophomonas* infection is not an opportunistic parasitic pathogen [12]. On the other hand, in the north of Iran, due to its geographical location, climate, and humidity, there are suitable conditions for the transmission and spread of parasites [17]. Increased awareness among healthcare professionals about the neglected *Lophomonas* infection may lead to earlier and more accurate diagnoses, facilitating prompt and effective treatment interventions and ultimately improving patient outcomes.

The radiological findings in our patient included alveolar opacities and a peripheral “tree‐in‐bud” pattern, observed in both lungs. These diverse and non‐specific imaging features underscore the importance of considering *Lophomonas* infection as part of the differential diagnosis for respiratory symptoms, particularly in regions where this parasite is prevalent.

Recently a comprehensive registry‐ based study showed the most common radiological findings in patients with lophomoniasis included tree‐in‐bud nodules, alveolar consolidation, bronchiectasis, and centrilobular nodules, similar our patient, which mostly seen in the right lung and its middle and lower lobes [18]. These imaging findings are non‐specific and can overlap with other respiratory infections, making diagnosis challenging without additional diagnostic methods like BAL for microscopic examination of *Lophomonas* trophozoites. However, the overlapping clinical symptoms of lophomoniasis with other respiratory diseases, combined with the absence of pathognomonic radiological and bronchoscopic findings, may lead to misdiagnosis and underestimation of this condition.

For the first time in Iran, *Lophomonas* spp. was detected within the guts of German cockroaches ( *Blattella germanica* ) trapped in hospitals in Mazandaran, northern Iran. The presence of cockroaches in hospitals may pose a potential risk to patients and healthcare personnel [19]. Public education regarding preventive strategies is crucial, particularly in maintaining proper hygiene practices and reducing exposure to areas with high populations of arthropods. Infection serves as a crucial reminder to explore alternative causes of respiratory symptoms in patients with existing respiratory conditions. By increasing awareness and enhancing diagnostic skills, healthcare practitioners can improve the detection and treatment of *Lophomonas* infections, finally leading to better patient outcomes. This infection is not well comprehended, and numerous characteristics of the parasite are still unidentified [12].

The analysis of registry data indicated that the prevalence of *Lophomonas* among the Iranian individuals was approximately 22% [6, 12]. The clinical manifestations of this infection indicate considerable overlap with those of various other respiratory infections, thereby complicating the diagnosis of lophomoniasis, particularly in instances where it co‐exists with other respiratory infections [12]. Clinicians should consider the possibility of other pathogen comorbidities, particularly when the patient's symptoms are unresolved. The most common diagnostic method for *Lophomonas* detection is direct microscopic examination of wet mounts and/or stained smears, but molecular techniques can also be used to enhance diagnostic accuracy and confirm cases, particularly in settings with less experienced personnel and/or when the parasite density in specimens is low [2, 6, 12].

While the overall incidence of co‐infection remains low, the similarity in clinical symptoms between TB and lophomoniasis may result in potential misdiagnosis or underdiagnosis of lophomoniasis, leading to complicated diagnosis. This necessitates a heightened awareness among healthcare providers to consider *Lophomonas* infection in patients presenting with respiratory symptoms typical of tuberculosis, especially in endemic areas where both infections are prevalent.

In conclusion, the report emphasises the importance of recognising the potential for *Lophomonas*/TB co‐infection in endemic areas and advocates for increased vigilance and improved diagnostic practices to address this emerging health concern. Based on our case experiences, we recommend that pulmonary lophomoniasis be considered in the differential diagnosis of tuberculosis, particularly in cases where patients do not respond to antibiotic treatment. Additionally, individuals with high‐risk epidemiological factors should be assessed for this disease. Studying such co‐infections offers insights into how different pathogens interact within a host with compromised cognitive function AD, potentially revealing new therapeutic targets or diagnostic approaches for managing complex infections alongside neurodegenerative diseases.

## Author Contributions

M.M. and E.S. were responsible for writing the manuscript. M.F. done a critical revision of the entire manuscript. All authors participated in the review process and approved the manuscript's final version.

## Ethics Statement

We declare that appropriate written informed consent was obtained for the publication of this manuscript and associated images.

## Conflicts of Interest

The authors declare no conflicts of interest.

## Data Availability

Data will be made available on request.
